# Synaptic Inputs to the Mouse Dorsal Vagal Complex and Its Resident Preproglucagon Neurons

**DOI:** 10.1523/JNEUROSCI.2145-19.2019

**Published:** 2019-12-04

**Authors:** Marie K. Holt, Lisa E. Pomeranz, Kevin T. Beier, Frank Reimann, Fiona M. Gribble, Linda Rinaman

**Affiliations:** ^1^Florida State University, Department of Psychology and Program in Neuroscience, Tallahassee, Florida 32306-4301,; ^2^Laboratory of Molecular Genetics, Howard Hughes Medical Institute, Rockefeller University, New York, New York 10065,; ^3^University of California at Irvine, Department of Physiology and Biophysics, Irvine, California 92697, and; ^4^Institute of Metabolic Science, Metabolic Research Laboratories, University of Cambridge, Cambridge, United Kingdom CB2 0QQ

**Keywords:** GLP1, nodose ganglion, pseudorabies, rabies, stress, viral tracing

## Abstract

Stress responses are coordinated by widespread neural circuits. Homeostatic and psychogenic stressors activate preproglucagon (PPG) neurons in the caudal nucleus of the solitary tract (cNTS) that produce glucagon-like peptide-1; published work in rodents indicates that these neurons play a crucial role in stress responses. While the axonal targets of PPG neurons are well established, their afferent inputs are unknown. Here we use retrograde tracing with cholera toxin subunit b to show that the cNTS in male and female mice receives axonal inputs similar to those reported in rats. Monosynaptic and polysynaptic inputs specific to cNTS PPG neurons were revealed using Cre-conditional pseudorabies and rabies viruses. The most prominent sources of PPG monosynaptic input include the lateral (LH) and paraventricular (PVN) nuclei of the hypothalamus, parasubthalamic nucleus, lateral division of the central amygdala, and Barrington's nucleus (Bar). Additionally, PPG neurons receive monosynaptic vagal sensory input from the nodose ganglia and spinal sensory input from the dorsal horn. Sources of polysynaptic input to cNTS PPG neurons include the hippocampal formation, paraventricular thalamus, and prefrontal cortex. Finally, cNTS-projecting neurons within PVN, LH, and Bar express the activation marker cFOS in mice after restraint stress, identifying them as potential sources of neurogenic stress-induced recruitment of PPG neurons. In summary, cNTS PPG neurons in mice receive widespread monosynaptic and polysynaptic input from brain regions implicated in coordinating behavioral and physiological stress responses, as well as from vagal and spinal sensory neurons. Thus, PPG neurons are optimally positioned to integrate signals of homeostatic and psychogenic stress.

**SIGNIFICANCE STATEMENT** Recent research has indicated a crucial role for glucagon-like peptide-1-producing preproglucagon (PPG) neurons in regulating both appetite and behavioral and autonomic responses to acute stress. Intriguingly, the central glucagon-like peptide-1 system defined in rodents is conserved in humans, highlighting the translational importance of understanding its anatomical organization. Findings reported here indicate that PPG neurons receive significant monosynaptic and polysynaptic input from brain regions implicated in autonomic and behavioral responses to stress, as well as direct input from vagal and spinal sensory neurons. Improved understanding of the neural pathways underlying the recruitment of PPG neurons may facilitate the development of novel therapies for the treatment of stress-related disorders.

## Abstract (precis)

Stress responses are coordinated by widespread neural circuits. Homeostatic and psychogenic stressors activate preproglucagon (PPG) neurons in the caudal nucleus of the solitary tract (cNTS) that produce glucagon-like peptide-1; published work in rodents indicates that these neurons play a crucial role in stress responses. While the axonal targets of PPG neurons are well established, their afferent inputs are unknown.

## Introduction

The brain continuously monitors and prioritizes competing needs of the organism, with adaptive physiologic and behavioral responses organized by central neural circuits. For example, actual or perceived threats to well-being result in a rapid increase in sympathetic outflow and a suppression of appetite (Ulrich-Lai and Herman, 2009; Holt and Trapp, 2016). Complex neural systems control these processes, with the caudal brainstem dorsal vagal complex (DVC) [comprising the area postrema (AP), nucleus of the solitary tract (NTS), and dorsal motor nucleus of the vagus (DMV)] playing a key role in both behavioral and physiological responses to homeostatic and psychogenic threats (Ulrich-Lai and Herman, 2009; Myers et al., 2017).

One neuropeptide expressed within the DVC, glucagon-like peptide-1 (GLP-1), acts centrally in rodents to produce stress-like effects, including suppressed food and water intake (Tang-Christensen et al., 1996; Larsen et al., 1997; Merchenthaler et al., 1999; McKay et al., 2014; Terrill et al., 2019), reduced reward associated with drugs of abuse (Erreger et al., 2012; Graham et al., 2013; Shirazi et al., 2013), and increased sympathetic outflow (Yamamoto et al., 2002, 2003; Baggio et al., 2017; Ghosal et al., 2017). GLP-1 is produced by preproglucagon (PPG) neurons within the caudal NTS (cNTS) and the adjacent intermediate reticular nucleus (IRT) (Larsen et al., 1997; Merchenthaler et al., 1999). In mice, chemogenetic activation of PPG neurons robustly suppresses food intake (Gaykema et al., 2017; Liu et al., 2017; Holt et al., 2019), and PPG/GLP-1 neurons and GLP-1 receptor signaling within the brain contribute to stress-induced hypophagia in mice (Williams et al., 2018; Holt et al., 2019; Terrill et al., 2019) and rats (Maniscalco et al., 2015). However, little is known regarding neural circuits that recruit PPG neurons in response to homeostatic and psychogenic threats.

PPG neurons project widely to spinal and brain nuclei implicated in controlling autonomic outflow and motivated behavior (Llewellyn-Smith et al., 2011, 2013, 2015; Gu et al., 2013). The activity of PPG neurons is modulated by interoceptive feedback, and electrophysiological evidence suggests that these neurons receive direct synaptic input from vagal sensory afferents (Hisadome et al., 2010). *In vitro*, mouse PPG neurons are activated by cholecystokinin, leptin, serotonin, and the cytokine interleukin-6 (Hisadome et al., 2010, 2011; Anesten et al., 2016; Holt et al., 2017). *In vivo*, GLP-1-positive PPG neurons are activated to express cFOS in rats after homeostatic challenges (including rapid intake of an unusually large meal, mechanical gastric distension, and malaise-inducing treatments, e.g., lithium chloride or lipopolysaccharide), and after acute cognitive stressors that suppress food intake (Rinaman, 1999; Vrang et al., 2003; Hayes et al., 2009; Maniscalco et al., 2015; Kreisler and Rinaman, 2016). PPG neurons also express cFOS in mice after acute restraint stress (Terrill et al., 2019), and either acute or chronic inhibition of PPG neurons leads to reduced satiation during intake of an unusually large meal (Holt et al., 2019). Given these findings, it seems clear that PPG neurons are part of a larger neural network that responds to both physiological and cognitive threats (Maniscalco and Rinaman, 2017; Holt et al., 2019).

Considering the evidence summarized above, we hypothesized that PPG neurons in the mouse cNTS receive synaptic input from vagal sensory neurons and from brain regions implicated in autonomic control and motivated behavior. While many published studies have revealed sources of axonal input to the DVC in rats (see, e.g., van der Kooy et al., 1984; Veening et al., 1984; Berthoud et al., 1990; Ruggiero et al., 1996), similar investigations in mice have not been reported, and no studies have sought to reveal sources of input specifically to PPG neurons. Here, we first reveal central sources of direct axonal input to the mouse DVC using a standard neural tracer [cholera toxin subunit b (CTb)], followed by retrograde tracing of polysynaptic and monosynaptic inputs specific to PPG neurons using Cre-conditional pseudorabies (PRV) and rabies virus (RABV) in *mGlu-Cre* transgenic mice. Finally, we show that neurons within a subset of DVC-projecting brain regions are activated to express cFOS in mice after acute restraint stress. Collectively, our results provide the first description of central neural inputs to the DVC in mice, including stress-responsive inputs, the first side-by-side anatomical analysis of data generated using conditional polysynaptic PRV and monosynaptic RABV tracing, and the first description of monosynaptic and polysynaptic inputs specific to PPG neurons. These findings significantly advance our understanding of circuits through which the DVC in general, and PPG neurons in particular, can be recruited by stress-related stimuli that impact autonomic outflow and motivated behavior.

## Materials and Methods

Experimental protocols were approved by the Florida State University Institutional Animal Care and Use Committee and were consistent with the U.S. Public Health Service's Policy on the Humane Care and Use of Laboratory Animals and the Guide for the Care and Use of Laboratory Animals.

### 

#### Animals

Male and female *mGlu-Cre/tdRFP* (*n* = 44) and *mGlu-YFP* (*n* = 12) transgenic mice were bred in house and used as adults. WT adult male C57BL/6J mice (*n* = 3) were obtained from The Jackson Laboratory and used as controls in the viral tracing studies. Mice had *ad libitum* access to water and Purina chow and were kept on a 12 h light/dark cycle. *mGlu-Cre* transgenic mice express Cre recombinase under the control of the glucagon promoter, allowing selective targeting of GLP-1-expressing PPG neurons (Parker et al., 2012; Anesten et al., 2016; Holt et al., 2019), which also express a Cre-conditional fluorescent reporter, tdRFP (Luche et al., 2007). *mGlu-YFP* transgenic mice express the yellow fluorescent protein (YFP) Venus under the control of the glucagon promoter, enabling visualization of PPG neurons based on YFP immunoreactivity (Reimann et al., 2008; Llewellyn-Smith et al., 2011). *mGlu-Cre* and *mGlu-YFP* mice were established as local colonies from transgenic animals received by Florida State University in 2013 from Cambridge (UK) on a C57BL/6 background. The original Cambridge *mGlu-Cre* and *mGlu-YFP* mice were generated in 2008 and 2005, respectively, and maintained for >20 generations before receipt by Florida State University. At Florida State University, both colonies have been maintained for >15 generations on a C57BL6 background.

#### Stereotaxic microinjections targeting the cNTS/DVC

Mice (24.8 ± 4.6 g, mean ± SD) were anesthetized using isoflurane (1%–3%, 1.5 ml/min in O_2_) and placed in a stereotaxic frame with the nose pointing downward to expose the dorsal surface of the neck and facilitate access to the caudal brainstem. Using a surgical microscope, an incision was made through the skin along the midline extending from the occipital crest to the first vertebra, and the underlying muscles were separated to expose the roof of the fourth ventricle caudal to the cerebellum. The meningeal layer was penetrated using a 30 G needle, and obex was visualized. To target the cNTS, the tip of a glass needle was inserted 400 μm lateral and 100 μm rostral to obex, and then lowered 350 μm below the dorsal surface of the brainstem. Viral titers and sources are listed in Table 1.

**Table 1.** List of viruses

Virus	Titer (pfu/ml)	Source	References
PRV-introvert-GFP	5.6 × 10^8^	Lisa E. Pomeranz, Rockefeller University, New York	Pomeranz et al., 2017
AAV5-EF1a-FLEX-TVA:mCherry	2.13 × 10^12^	GVC-AAV-67, Stanford Gene Vector and Virus Core	Watabe-Uchida et al., 2012
AAV8/733-CAG-FLEX-RabiesG	2.4 × 10^13^	GVC-AAV-59, Stanford Gene Vector and Virus Core	Watabe-Uchida et al., 2012
(EnvA)-RV-ΔG-GFP	2 × 10^8^	Kevin Beier, University of California at Irvine, Irvine, CA	Wickersham et al., 2007

##### Experiment 1.

To map all central sources of input to the cNTS/DVC in a non–cell type-specific manner, *mGlu-YFP* mice (*n* = 3, all male) received bilateral microinjections of CTb (Luppi et al., 1990) (100 nl, 0.1%). Mice were transcardially perfused 4 d later, as described below (see Transcardial perfusion and tissue preparation).

##### Experiment 2.

PRV-Introvert-GFP was developed by Pomeranz et al. (2017) to enable Cre-dependent viral replication, retrograde transsynaptic transport, and expression of GFP. Expression of both GFP and viral thymidine kinase, which is required for PRV replication and propagation, occurs only after an initial Cre-mediated recombination event. Once recombination occurs, the virus continues to replicate, express GFP, and move across synapses in the retrograde direction without requiring Cre. To map polysynaptic inputs specific to PPG neurons within the cNTS, *mGlu-Cre/tdRFP* mice (*n* = 27, 13 female) and WT control mice (*n* = 3, all male) received unilateral cNTS-targeted microinjections of 100–400 nl of a 3:1 mix of PRV-Introvert-GFP (Pomeranz et al., 2017) and CTb (0.1%) or PRV-Introvert-GFP on its own. These 27 mice are a subset of 32 mice used in the experiment. Of these, 11 were injected with the smallest volume (100 nl) of the PRV-Introvert-PRV and CTb mixture. Five of these mice (5 of 11) displayed CTb labeling but no PRV-GFP labeling. In the other 6 mice, the 100 nl injection volume generated PRV-GFP labeling that did not differ from labeling in mice that were injected with larger volumes (up to 400 nl; *n* = 21) when postinoculation time points were matched. Thus, PRV-GFP labeling data from 27 mice (excluding the 5 with no PRV-GFP labeling) were pooled at each postinoculation time point, regardless of injection volume. Mice were transcardially perfused 22 h (*n* = 3), 48 h (*n* = 6), 72 h (*n* = 9), 96 h (*n* = 4), or 120 h (*n* = 5) postinoculation.

##### Experiment 3.

For monosynaptic retrograde tracing from cNTS PPG neurons, bilateral injections of 300 nl of a 1:1 mixture of AAV5-EF1a-FLEX-TVA:mCherry and AAV8/733-CAG-FLEX-RabiesG (Table 1) were targeted to the cNTS of *mGlu-Cre/tdRFP* transgenic mice (*n* = 3, 2 female). In a second surgery 24 d later, a unilateral injection of with 500 nl (EnvA)-RABV-ΔG-GFP (Table 1) was targeted to the cNTS and distributed in two medial-lateral injection sites: (1) 250 μm lateral, 100 μm rostral, and 450–350 μm below the surface of the brainstem; and (2) 400 μm lateral, 100 μm rostral, and 450–350 μm below the surface of the brainstem. Mice were transcardially perfused 7 d later.

##### Experiment 4.

To assess stress-induced activation of neurons projecting to the cNTS/DVC, *mGlu-YFP* transgenic mice (*n* = 9, 7 male) received bilateral iontophoretic delivery of CTb (5 min, 7 s on/off, 5 μA) targeted to the DVC. Seven days later, mice assigned to the stress condition (*n* = 4) were exposed to 30 min acute restraint in a mouse decapicone (MDC-200, Braintree Scientific) as previously described (Holt et al., 2019; Terrill et al., 2019). Restraint stress was conducted 4 h after lights on. Control mice (*n* = 5) were left undisturbed in their home cages. Mice were transcardially perfused 60 min after the end of restraint stress, or at a similar time of day for controls.

#### Transcardial perfusion and tissue preparation

Mice were anesthetized using pentobarbital sodium (Fatal Plus, 100 mg/kg, i.p.; Henry Schein) and then transcardially perfused with ice-cold PB (0.1 m, pH 7.2) followed by 4% formaldehyde in PB. The brain was extracted and placed in 4% formaldehyde in PB at 4°C. The nodose ganglia (NG) were postfixed *in situ* overnight in 4% formaldehyde in PB at 4°C before extraction. Brains and NG were cryoprotected for at least 24 h in 20% sucrose at 4°C. Using a freezing microtome, brains were sectioned into four series of 40-μm-thick coronal sections, such that each series contained a complete set of rostrocaudal sections spaced by 160 μm, from the caudal medulla through the frontal cortex. Sections were processed as described below (see Immunoperoxidase and immunofluorescence labeling). Intact NG were first imaged on a confocal microscope (see Light microscopy), then sectioned on a cryostat (25 μm). NG sections were collected onto Superfrost Plus microscope slides (Fisherbrand #12-550-15, Thermo Fisher Scientific) and stored at −20°C before immunoprocessing.

#### Immunoperoxidase and immunofluorescence labeling

Coronal brain sections were processed to immunolabel a variety of antigens, depending on the experiment (Tables 2, 3). Sections were removed from cryoprotectant, rinsed in four changes of 0.1 m PB over 1 h, and pretreated with 0.5% sodium borohydride for 20 min at room temperature. For immunoperoxidase, sections were also pretreated with 0.15% hydrogen peroxide for 15 min at room temperature to suppress endogenous peroxidase activity. After rinsing, sections were incubated overnight at room temperature in primary antibody (Table 2) diluted in 0.1 m PB containing 0.3% Triton-X and 1% normal donkey serum. Sections were rinsed in four changes of 0.1 m PB and then incubated for 1–2 h at room temperature in a 1:500 dilution of biotin- or fluorophore-conjugated affinity purified donkey secondary antibody (Table 2) in 0.1 m PB containing 0.3% Triton-X and 1% normal donkey serum. After four rinses in 0.1 m PB for 1 h, sections were either mounted onto glass slides (for immunofluorescence) or incubated using Vectastain Elite avidin-biotin complex kit reagents diluted in 0.1 m PB containing 0.3% Triton for 1.5–2 h (for immunoperoxidase). Peroxidase activity was localized using diaminobenzidine catalyzed with hydrogen peroxide. Following mounting, sections were dehydrated in increasing concentrations of ethanol, cleared in xylene, and coverslipped using Cytoseal 60 (Electron Microscopy Sciences, 18007). Slide-mounted NG sections were processed for immunofluorescence labeling of GFP and TH (tyrosine hydroxylase), as described above.

**Table 2.** List of antibodies

Target	Source and catalog #	Antibody registry accession #	Dilution	Secondary antibody
GFP	Abcam, Ab13790	AB_300798	1:5000 (IF), 1:20,000 (IHC)	AlexaFluor-488 anti-chicken (IF), biotin-conjugated anti-chicken (IHC)
CTb	List #703	AB_10013220	1:2000 (IF), 1:20,000 (IHC)	Cy3 anti-goat (IF), biotin-conjugated anti-goat (IHC)
tdRFP/mCherry	Takara Bio, #632496	AB_10013483	1:2000	Cy3 anti-rabbit
PRV	Rb133, noncommercial	AB_2315209	1:2000	AlexaFluor-647 anti-rabbit
TH	Millipore, AB152	AB_390204	1:2000	AlexaFluor-647 anti-rabbit
AVP	Millipore, AB1565	AB_90782	1:1000	AlexaFluor-647 anti-rabbit
OT	Peninsula, T-4084	AB_518524	1:1500	AlexaFluor-647 anti-rabbit
cFOS	Cell Signaling Technology, 9F6	AB_2247211	1:20,000	Biotin-conjugated anti-rabbit (IHC)

**Table 3.** Experimental design

Experiment	Injection	*N* (male, female)	Tissue analysis
Inputs to the mouse DVC	CTb (100 nl)	3 (3, 0)	IHC: CTb
Monosynaptic and polysynaptic inputs to cNTS PPG neurons	PRV (100–400 nl) + CTb (25–100 nl)	30 (17, 13)	IHC/IF: PRV, CTb, tdRFP, GFP, AVP, TH, OT
Monosynaptic inputs to cNTS PPG neurons	RABV (500 nl)	3 (1, 2)	IF: GFP, tdRFP, TH; RNAscope: *Crh*, *Gad1*
Stress-induced activation of DVC-projecting neurons	CTb (iontophoresis, 5 min; 7 s off/on)	9 (7, 2)	IHC: CTb, cFOS

#### RNAscope ISH

Tissue sections from a subset of *mGlu-Cre/tdRFP* mice used for monosynaptic RABV tracing were processed using FISH to reveal mRNA transcripts expressed by RABV-infected (GFP-immunopositive) neurons. mRNA for corticotropin releasing hormone (*Crh*) or glutamic acid decarboxylase 1 (*Gad1*) was detected using RNAscope Multiplex Fluorescent Reagent Kit version 2 (Advanced Cell Diagnostics, #323100). Coronal sections containing Barrington's nucleus (Bar) or AP were pretreated with hydrogen peroxide (Advanced Cell Diagnostics, #322335) for 30 min at room temperature and slide-mounted in dH_2_O. Following 10 s dehydration in 100% ethanol, a hydrophobic barrier was created around 3 or 4 sections, which were then treated with proteinase IV (Advanced Cell Diagnostics, #322336) for 25 min at room temperature. After three rinses in dH_2_O, sections were left to incubate in RNAscope Probe Mm-Crh-C1 (Advanced Cell Diagnostics, #316091-C1) or Mm-Gad1-C2 (Advanced Cell Diagnostics, #500951-C2) for 2 h at 40°C in an HybEZTM oven (Advanced Cell Diagnostics) followed by three amplification steps, and labeling of the Mm-Crh probe with Cy3-conjugated Tyramine Signal Amplification Plus (PerkinElmer) according to the Advanced Cell Diagnostics protocol. Sections were washed in wash buffer (Advanced Cell Diagnostics, #310091) 3 × 3 min between steps. The same sections were subsequently immunolabeled for GFP (to detect RABV-infected cells) and TH as described above. Following immunolabeling, sections were dehydrated in increasing concentrations of ethanol, cleared in xylene, and coverslipped using Cytoseal 60 (Electron Microscopy Sciences, 18007).

#### Light microscopy

Images of GFP, cFOS, and CTb immunoperoxidase labeling were captured on a Keyence microscope (BZ-X700). Fluorescence immunolabeling and ISH were visualized on a Leica Microsystems TCS SP8 confocal microscope using a 20× air objective and a 40× or 100× oil-immersion objective. AlexaFluor-488, Cy3, and AlexaFluor-647 were excited using a 488 nm OPSL, 552 nm OPSL, and 638 nm diode laser, respectively. Images were acquired sequentially using Leica Microsystems LAS 4.0 image collection software. Brightness and contrast were adjusted using Fiji open source biological image analysis software (Schindelin et al., 2012).

#### Experimental design and statistical analysis

Experiments were designed as indicated in Table 3. For polysynaptic tracing studies with PRV-Introvert-GFP, mice were assigned to one of nine different surgical sessions, and then anesthetized and perfused at various postinoculation intervals. To quantify GFP labeling densities within individual brain regions at the 96 h postinoculation interval, specific brain ROIs were outlined in cases with maximal GFP labeling in that ROI, and the same ROI outline was then applied to the same brain regions across animals. Using FIJI (Schindelin et al., 2012), the area within each ROI that contained GFP labeling that was more intense than a standardized, preset threshold was measured, and reported as a percentage of the total ROI area. For stress-induced cFOS expression, the person counting cells was blind to the experimental condition. CTb- and cFOS-positive neurons were counted in a predetermined subset of brain regions that (in other mice) displayed monosynaptic RABV-labeled inputs to cNTS PPG neurons. Cells were counted as cFOS-positive if black nuclear labeling was visible, regardless of labeling intensity. Within each brain ROI, counts of CTb- and/or cFOS-positive neurons in stressed versus nonstressed control mice were compared using multiple unpaired Student's *t* test, corrected for false discovery rate (Benjamini and Hochberg, 1995; Glickman et al., 2014) (for abbreviations, see Table 4).

**Table 4.** Brain region and landmark abbreviations

Abbreviation	Region
3V	Third ventricle
4V	Fourth ventricle
aca	Anterior commissure
AP	Area postrema
Arc	Arcuate nucleus
Aq	Aqueduct
Bar	Barrington's nucleus
BLA	Basolateral amygdala
BMA	Basomedial amygdala
BST	Bed nucleus of the stria terminalis
CC	Central canal
CeA	Central amygdala
CeL	Central amygdala, lateral part
CeM	Central amygdala, medial part
cNTS	Nucleus of the solitary tract, caudal part
cp	Cerebral peduncle
DH	Spinal cord dorsal horn
DMV	Dorsal motor nucleus of the vagus
DR	Dorsal raphe nucleus
DVC	Dorsal vagal complex
fx	Fornix
Gi	Gigantocellular nucleus
GiA	Gigantocellular nucleus, anterior part
dHPF	Hippocampal formation, dorsal part
vHPF	Hippocampal formation, ventral part
IRT	Intermediate reticular nucleus
KF	Kölliker-Fuse nucleus
LC	Locus ceruleus
LH	Lateral hypothalamus
LS	Lateral septum
LV	Lateral ventricle
MCN	Medial cerebellar nucleus
MnR	Median raphe
NG	Nodose ganglion
NTS	Nucleus of the solitary tract
cNTS	Nucleus of the solitary tract, caudal part
opt	Optic tract
PAG	Periaqueductal gray
vlPAG	Periaqueductal gray, ventrolateral part
PBN	Parabrachial nucleus
PFC	Prefrontal cortex
PHA	Posterior hypothalamic area
Pir	Piriform cortex
POA	Preoptic area
PSTh	Parasubthalamic nucleus
PVN	Paraventricular nucleus of the hypothalamus
PVT	Paraventricular nucleus of the thalamus
py	Pyramidal tract
RLi	Rostral linear raphe nucleus
RMg	Raphe magnus
RPa	Raphe pallidus
S1	Somatosensory cortex
scp	Superior cerebellar peduncle
Sp5	Spinal trigeminal nucleus
SuM	Supramammillary nucleus
VMH	Ventromedial hypothalamus
VTA	Ventral tegmental area

## Results

### Experiment 1: central inputs to the mouse DVC

**Figure 1.**
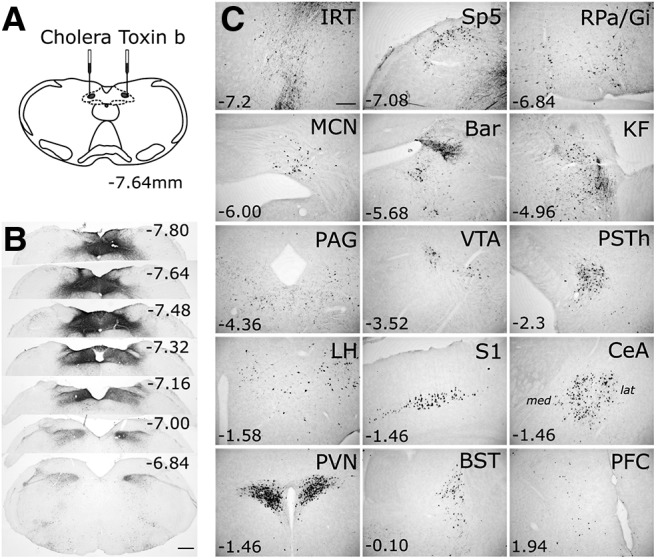
Central inputs to the DVC in mice revealed using CTb. ***A***, Schematic illustrating bilateral injections of 100 nl CTb targeted to the cNTS, which produced labeling throughout the DVC (evident in ***B***). ***B***, Representative images of CTb immunoperoxidase labeling at the DVC injection site. Distance from bregma (in mm) is indicated for each coronal section. Scale bar, 400 μm. ***C***, Representative images of retrogradely transported CTb in higher brain regions. Distance from bregma (in mm) is indicated for each region. For the CeA image: *med*, Medial CeA; *lat*, lateral CeA. Other abbreviations are defined in Table 4. Scale bar: IRT, 200 μm (applies to all other images in ***C***).

To identify brain regions that provide direct axonal input to the mouse DVC, we targeted bilateral microinjections of CTb neural tracer to the cNTS (Fig. 1*A*). A representative injection site is shown in Figure 1*B*. Retrograde CTb neural labeling was detected in multiple brainstem, midbrain, and forebrain areas (Fig. 1*C*; Table 5). Particularly dense clusters of retrogradely labeled neurons were present within the IRT, Bar, Kölliker-Fuse nucleus (KF), parasubthalamic nucleus (PSTh), medial and lateral subregions of the central amygdala (CeA), paraventricular nucleus of the hypothalamus (PVN), and lateral hypothalamus (LH). Most retrogradely labeled neurons within the CeA occupied its medial subnucleus, with a smaller number of neurons present in its lateral subnucleus (Fig. 1*C*). Since only male mice were included in the CTb tracing study, potential sex differences in central neural inputs to the DVC were not assessed. However, no sex differences were evident in results from the PRV or RABV tracing experiments, described further below.

**Table 5.** List of central inputs to the DVC and PPG neurons in the NTS

Region	CTb	PRV-GFP (96 h)	PRV-GFP (120 h)	RABV-GFP
Telencephalon				
Prelimbic cortex	+	−	√	−
Infralimbic cortex	+	−	√	−
Preoptic area	−	−	√	−
Insular cortex	+	−	−	−
Lateral septum	−	−	√	−
Bed nucleus of the stria terminalis	++	√	√	√
Central amygdala	+++	√	√	√
Basomedial amygdala	−	−	√	−
Somatosensory cortex	++	−	−	−
Diencephalon				
Paraventricular hypothalamus	+++	√	√	√
Piriform cortex	−	−	√	−
Ventromedial hypothalamus	−	−	√	−
Lateral hypothalamus	+++	√	√	√
Arcuate nucleus	+	−	√	−
Ventral hippocampus	−	−	√	−
Dorsal hippocampus	−	−	√	−
Paraventricular thalamus	−	−	√	−
Parasubthalamic nucleus	+++	√	√	√
Mesencephalon				
Supramammillary nucleus	−	−	√	−
Deep mesencephalic nucleus	+	−	−	−
Ventral tegmental area	++	−	√	−
Rostral linear raphe	++	−	√	−
Median raphe	−	−	√	−
Dorsal raphe	−	−	√	−
Dorsolateral periaqueductal gray	+	√	√	−
Lateral periaqueductal gray	++	√	√	√
Ventrolateral periaqueductal gray	++	√	√	√
Brainstem				
Kölliker-Fuse nucleus	+++	√	√	√
Parabrachial nucleus	−	−	√	−
Barrington's nucleus	+++	√	√	√
Locus ceruleus	−	−	√	−
Anterior gigantocellular nucleus	++	√	√	√
Raphe pallidus	+	−	√	√
Raphe magnus	+	−	√	√
Medial cerebellar nucleus	++	−	−	−
Lateral posterior gigantocellular nucleus	+++	√	√	√
Ventral gigantocellular nucleus	+	√	√	√
Medial vestibular nucleus	+	−	−	−
Superior vestibular nucleus	+	−	−	−
Raphe obscurus	+	−	−	−
Spinal trigeminal nucleus	++	√	√	−
Area postrema	NA	√	√	√
Intermediate reticular nucleus	+++	√	√	√

+, A few CTb-positive neurons; ++, Moderate numbers of CTb-positive neurons; +++, Large numbers of CTb-positive neurons; −, No detectable CTb-positive neurons; √, GFP-labeled neurons; NA, close proximity of region to DVC injection site precludes analysis of retrograde CTb labeling.

### Experiment 2: Cre-dependent PRV-Introvert-GFP reveals local and distant central sources of synaptic input to PPG neurons

**Figure 2.**
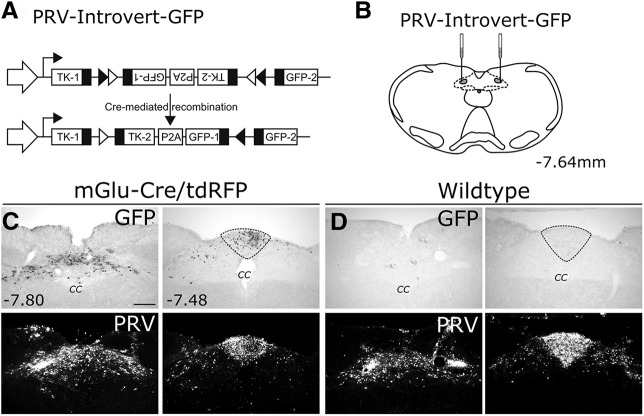
PRV-Introvert-GFP replicates and expresses GFP in a Cre-dependent manner. ***A***, Schematic illustrating the basis for Cre dependence of PRV-Introvert-GFP, as described in Materials and Methods. ***B***, Schematic illustrating bilateral microinjections of PRV-introvert-GFP targeted to the cNTS. TK: Thymidine kinase. ***C***, ***D***, Immunoperoxidase labeling of GFP expressed by neurons infected with PRV-Introvert-GFP (top row) and immunofluorescence staining of PRV (bottom row) at two rostrocaudal levels (distances from bregma are indicated in mm) in *mGlu-Cre/tdRFP* (***C***) and WT mice (***D***). The absence of GFP labeling in WT mice (***D***) indicates the Cre dependence of GFP expression in *mGlu-Cre/tdRFP* mice (***D***). Scale bar: (in ***C***) ***C***, ***D***, 200 μm. The AP is outlined.

To determine whether brain regions that project to the DVC include neurons that are directly or indirectly presynaptic to PPG neurons, a Cre-dependent PRV of the Bartha strain, PRV-Introvert-GFP (Pomeranz et al., 2017) (Fig. 2*A*), was microinjected bilaterally into the DVC of *mGlu-Cre/tdRFP* transgenic mice (Fig. 2*B*). GFP labeling was evident 72 h after inoculation within the cNTS and adjacent AP (Figs. 2*C*, top row, 3*A*); conversely, GFP labeling was completely absent at the same 72 h survival time in WT mice that were similarly injected with PRV-Introvert-GFP (Fig. 2*D*, top row). Similar to results in *mGlu-Cre/tdRFP* mice, PRV immunolabeling in WT mice was observed within the DVC, including the AP (Fig. 2*C*,*D*, bottom row), and in other brain regions that project directly to the cNTS/DVC injection site (data not shown, but similar to CTb retrograde tracing shown in Fig. 1). Similar to CTb and other non–cell-type-specific monosynaptic retrograde tracers, viral replication is not necessary for PRV to be taken up by axon terminals within the injection site and transported retrogradely to neurons whose axon terminals occupy the injection site (Pomeranz et al., 2017). However, in the absence of Cre, neither PRV replication (necessary for viral capsid envelopment and transsynaptic transport) (Card et al., 1993) nor GFP expression occurs (Fig. 2*D*).

**Figure 3.**
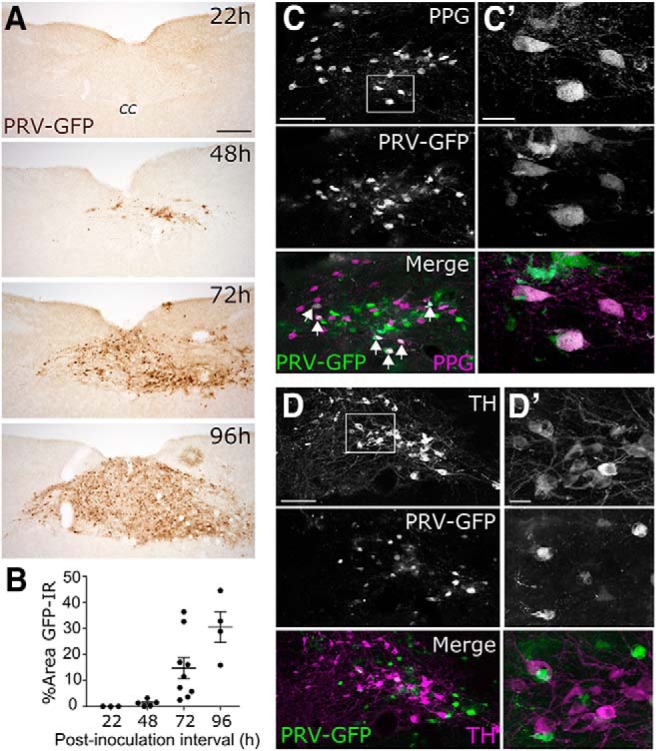
PPG neurons receive local synaptic input from within the DVC. ***A***, Representative images of GFP immunoperoxidase labeling within the cNTS (−7.6 mm from bregma) of transgenic *mGlu-Cre/tdRFP* mice terminated 22, 48, 72, or 96 h after inoculation with PRV-Introvert-GFP. Scale bar, 200 μm (applies to all panels in ***A***). ***B***, GFP labeling densities within the cNTS in mice killed at each postinoculation interval. Individual data points as well as summary data (mean ± SEM) are shown. ***C***, Immunofluorescence images of PPG neurons (tdRFP detected with anti-dsRed antibody, magenta) and GFP labeling (green) expressed by neurons infected with PRV-Introvert-GFP in a mouse killed at the 48 h postinoculation time point. Double-positive cells are indicated with white arrows in the merged image in ***C***, and a few are shown at higher magnification in ***C′***. Scale bars: ***C***, 100 μm; ***C′***, 10 μm. ***D***, Immunofluorescence images of TH (magenta) and GFP labeling (green) within the cNTS 48 h after PRV-Introvert-GFP injection. None of the GFP-positive infected neurons is TH-positive. Scale bar: ***D***, 100 μm. Higher-magnification images of inset are shown on the right (***D′***). Scale bar: ***D′***, 10 μm.

#### Time course of PRV-Introvert-GFP replication and transport

The time course of GFP expression was examined in additional *mGlu-Cre/tdRFP* mice killed 22, 48, 72, or 96 h after unilateral injection of PRV-Introvert-GFP targeted to the DVC.

#### DVC

GFP labeling was absent within the DVC at 22 h after inoculation, was first observed at 48 h, and continued to increase at the 72 and 96 h time points (Fig. 3*A*,*B*). At 48 h, a subset of tdRFP-positive PPG neurons (detected with anti-dsRed antibody) were double-labeled for GFP, evidence for successful Cre-mediated recombination of PRV-Introvert-GFP viral DNA (Fig. 3*C*,*C′*). Non-PPG cNTS neurons were also GFP-positive (Fig. 3*C*,*D*), evidence that these neurons provide local synaptic input to infected PPG neurons. Their distribution within the cNTS suggested that they might include the A2 noradrenergic cell group; however, noradrenergic (i.e., TH-positive) cNTS neurons were not observed to be double-labeled for GFP (Fig. 3*D*,*D′*).

#### Higher brain regions

**Figure 4.**
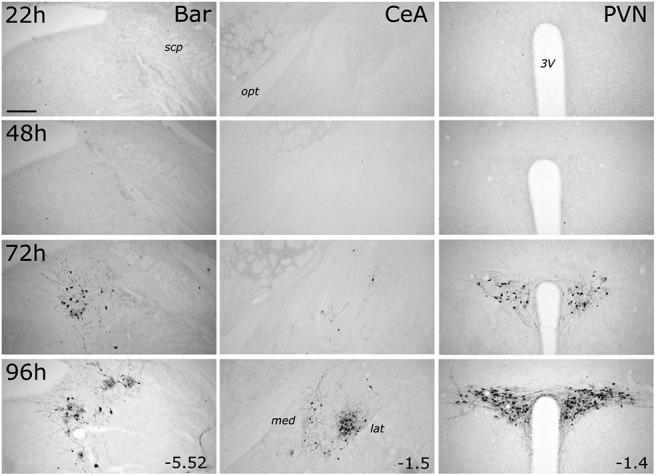
PRV-Introvert-GFP-induced expression of GFP increases with postinoculation interval in Bar, PVN, and CeA. GFP immunoperoxidase labeling in Bar (left column), CeA (middle column), and PVN (right column) in transgenic *mGlu-Cre/tdRFP* mice terminated 22, 48, 72, or 96 h after inoculation. For each brain region, distance from bregma (in mm) is indicated. Abbreviations are defined in Table 4. Scale bar, Top left, 200 μm (applies to all images).

**Figure 5.**
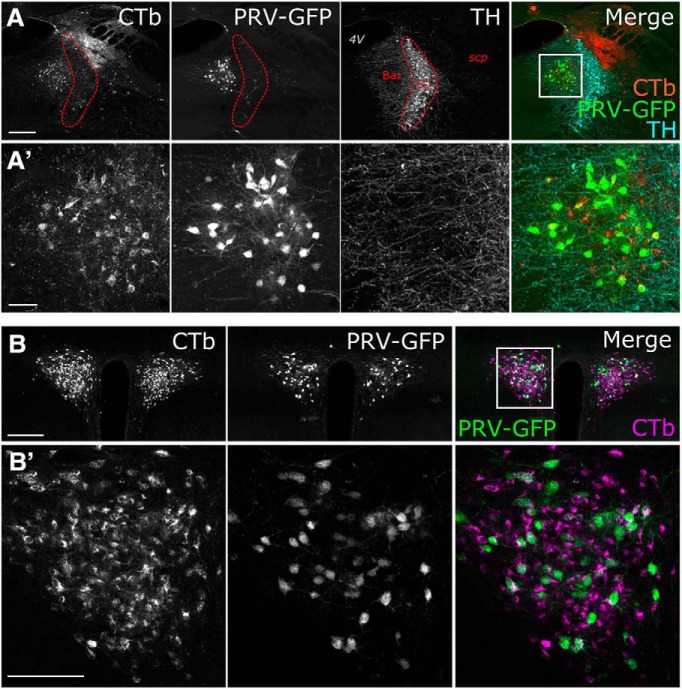
A subset of DVC-projecting PVN and Bar neurons are synaptically linked to cNTS PPG neurons. Immunofluorescence labeling for CTb and GFP within Bar (***A***) and the PVN (***B***) 96 h after microinjection of a mixture of PRV-Introvert-GFP and CTb into the cNTS. Images of immunolabeling in Bar (***B***) also include TH immunolabeling (cyan) to identify the LC. ***A′***, ***B′***, Higher-magnification images of the boxed regions. Scale bars: ***A***, ***B***, 100 μm; ***A′***, ***B′***, 50 μm.

GFP labeling within the pontine Bar, the telencephalic CeA, and the hypothalamic PVN was first detected at 72 h after inoculation, and was more pronounced at 96 h (Fig. 4). Within the CeA, GFP-positive neurons were clustered in its lateral subnucleus, with only a few labeled neurons present in the medial CeA at the 96 h time point (Fig. 4). Bar was easily distinguishable from the anatomically adjacent locus ceruleus (LC), which contains a dense population of TH-positive noradrenergic neurons (Fig. 5*A*). Many (but not all) GFP-positive cells within Bar and PVN also were immunopositive for CTb (which was coinjected with PRV-Introvert-GFP into the cNTS), whereas many CTb-positive neurons were not GFP-positive (Fig. 5*A*,*B*). While this result might reflect reduced efficiency of PRV/GFP labeling versus CTb labeling, it is consistent with our expectation that only a subset of DVC-projecting neurons in Bar and PVN impinge directly or indirectly onto infected PPG neurons in the cNTS. Within the PVN, one GFP-labeled neuron was immunopositive for oxytocin (OT), and one was immunopositive for vasopressin (AVP); however, the large majority of infected PVN neurons were not double-labeled (data not shown).

**Figure 6.**
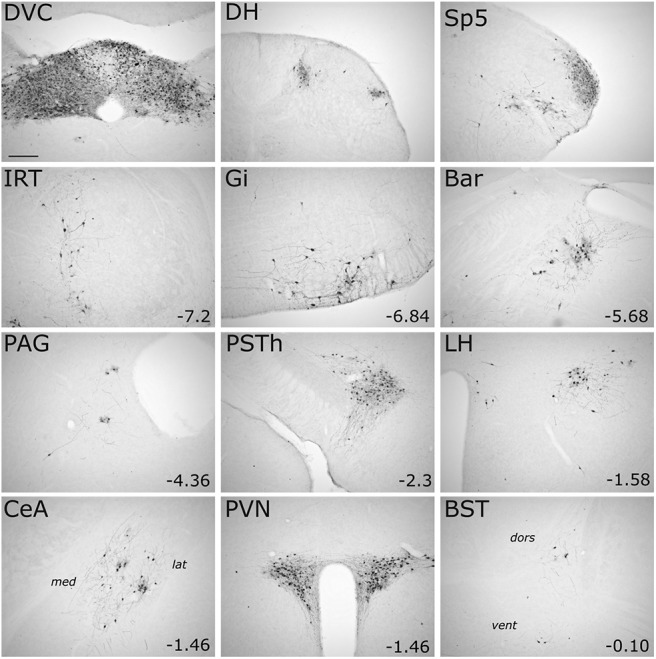
Brain circuits impinging on cNTS PPG neurons revealed with PRV-Introvert-GFP. Representative images of GFP immunoperoxidase labeling in different brain areas of a *mGlu-Cre/tdRFP* mouse 96 h after a microinjection of PRV-Introvert-GFP was targeted to the cNTS. For each brain region, distance from bregma (in mm) is indicated. For the CeA image: *med*, Medial CeA; *lat*, lateral CeA. For the BST image: *dors*, lateral BST dorsal to the anterior commissure; *vent*, lateral BST ventral to the anterior commissure. Other abbreviations are defined in Table 4. Scale bar: Top left, 200 μm (applies to all panels).

**Figure 7.**
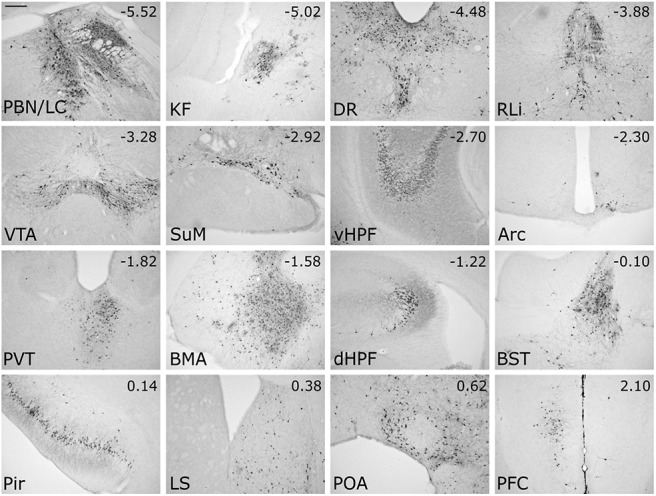
Longer postinoculation intervals reveal additional labeling of first-order and/or higher-order inputs to cNTS PPG neurons. Representative images of brain areas containing GFP immunoperoxidase labeling 120 h after cNTS microinjection of PRV-Introvert-GFP. For each brain region, distance from bregma (in mm) is indicated. Abbreviations are defined in Table 4. Scale bar: Top left, 200 μm (applies to all panels).

In addition to Bar, CeA, and PVN, GFP immunolabeling was evident within the spinal cord dorsal horn and other brainstem, midbrain, and forebrain regions at 96 h after inoculation (Fig. 6; Table 5). Although PRV-Introvert-GFP was targeted unilaterally to the cNTS/DVC, labeling in most brain regions was bilateral; exceptions included the CeA, BST, PSTh, Bar, and IRT. At the longest postinoculation interval (120 h), additional brain regions were observed to contain GFP-positive, but not CTb-positive, cells (Fig. 7; Table 5), suggesting that those regions do not directly target the DVC, but provide indirect input to infected PPG neurons in the cNTS. These regions included the parabrachial nucleus, LC, dorsal raphe, supramammillary nucleus, dorsal and ventral hippocampus, paraventricular thalamus, basomedial amygdala, and lateral septum (Fig. 7; Table 5).

**Figure 8.**
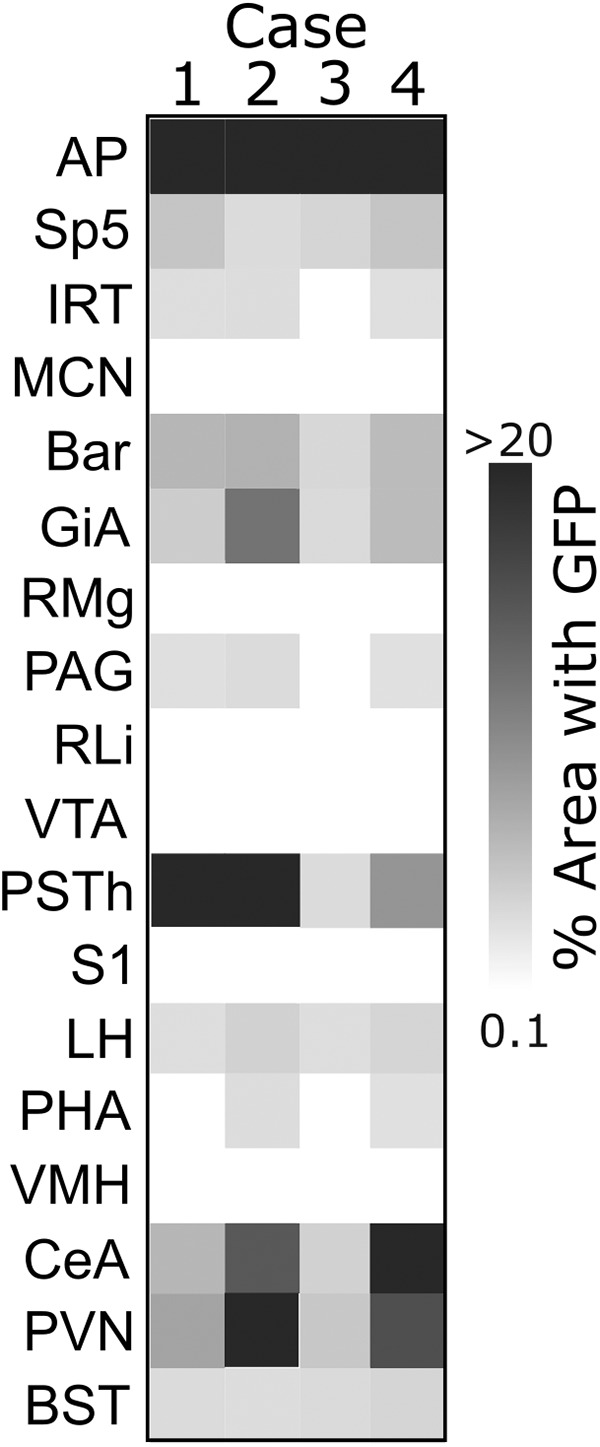
Quantification of retrograde viral labeling in brain regions impinging on PPG neurons. GFP immunoperoxidase labeling density was quantified in *mGlu-Cre/tdRFP* (*n* = 4) and WT (*n* = 3) mice injected with PRV-Introvert-GFP into the cNTS, and killed 96 h after inoculation. Darker shades represent higher densities of GFP labeling in *mGlu-Cre/tdRFP* mice. No GFP labeling was detected in any brain region in WT mice. Abbreviations are defined in Table 4.

Focusing on sources of input identified at 96 h after inoculation, we quantified the density of GFP immunolabeling in several brain ROIs that were distributed along the rostrocaudal axis (Fig. 8). The densest GFP labeling was found in the PVN, lateral CeA, PSTh, and AP of *mGlu-Cre/tdRFP* mice; no GFP labeling was detected in any brain region in WT mice (data not shown). Interestingly, several brain regions that project directly to the DVC (based on CTb labeling; Fig. 1*A*) contained little or no detectable GFP labeling 96 h after inoculation. These regions included the medial cerebellar nucleus (MCN), raphe magnus (RMg), raphe pallidus (RPa), rostral linear raphe, VTA, and somatosensory cortex (S1), suggesting that neurons within these regions project to the DVC but do not provide direct or indirect synaptic input to cNTS PPG neurons.

### Experiment 3: monosynaptic input to PPG neurons

**Figure 9.**
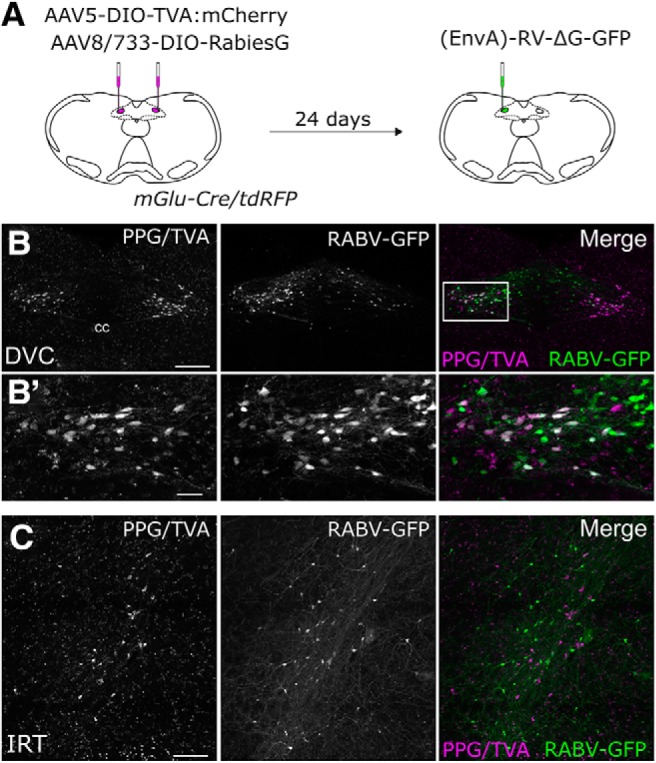
Monosynaptic, retrograde RABV tracing from cNTS PPG neurons. ***A***, Schematic illustrating bilateral microinjections of a mixture of AAV5-DIO-TVA:mCherry and AAV8:733-DIO-RabiesG into the cNTS of *mGlu-Cre/tdRFP* transgenic mice, followed 24 d later by unilateral cNTS microinjection of (EnvA)-RABV-ΔG-GFP. ***B***, Immunofluorescence images of PPG “starter” neurons within the cNTS, defined by their coexpression of tdRFP and TVA:mCherry (detected with anti-dsRed antibody, magenta) and GFP (green) expressed after successful complementation/replication of (EnvA)-RABV-ΔG-GFP. ***B′***, Higher-magnification images. Scale bars: ***B***, 200 μm; ***B′***, 40 μm. ***C***, Immunofluorescence images of PPG neurons within the IRT (detected with anti-dsRed antibody, magenta, which also would label TVA if it was expressed by the same cells). Although GFP-positive, monosynaptic RABV-infected neurons were present within the IRT (middle); none of the infected IRT neurons was immunopositive for dsRed, evidence that none of these was PPG neurons; and none expressed TVA, as expected given the cNTS viral injection site. Scale bar, 100 μm.

**Figure 10.**
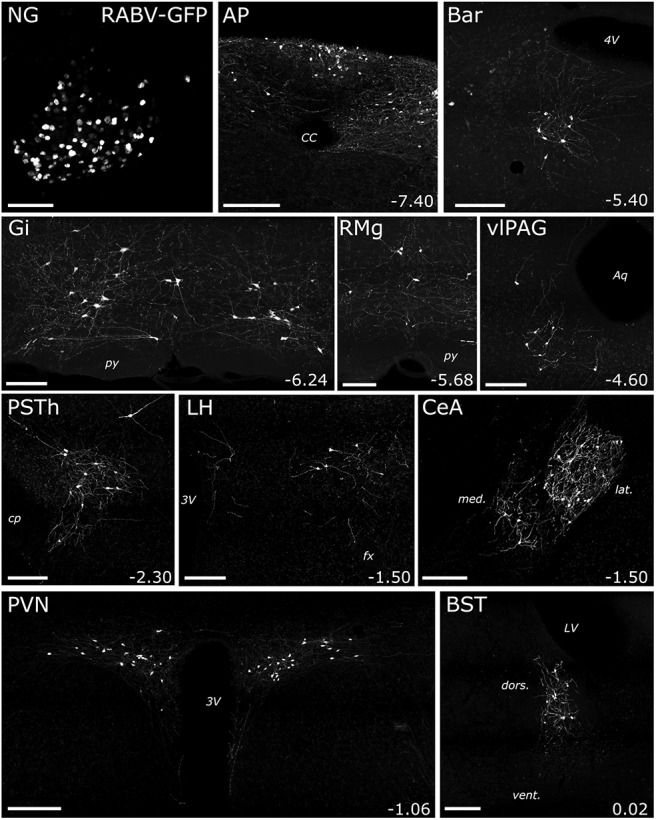
Additional peripheral (NG) and central sources of monosynaptic input to cNTS PPG neurons. GFP immunofluorescence labeling in tissue from a representative mouse is depicted, 7 d after microinjection of (EnvA)-RABV-ΔG-GFP targeted to the left cNTS. For the CeA image: *med*, Medial CeA; *lat*, lateral CeA. For the BST image: *dors*, lateral BST dorsal to the anterior commissure; *vent*, lateral BST ventral to the anterior commissure. For each brain region, distance from bregma (in mm) is indicated. Abbreviations are defined in Table 4. Scale bars, 200 μm.

**Figure 11.**
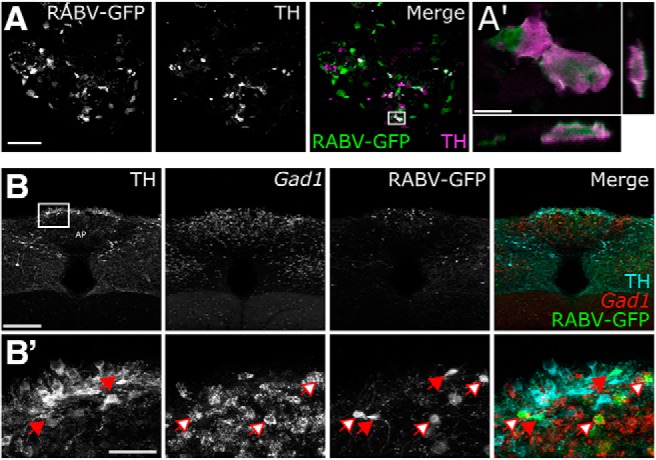
Phenotypes of RABV-infected NG and AP neurons providing direct synaptic input to cNTS PPG neurons. ***A***, GFP (green) and TH immunolabeling (magenta) in the left NG 7 d after inoculation with (EnvA)-RABV-ΔG-GFP into the left cNTS. Scale bar: ***A***, 100 μm. ***A***, Boxed area (merge) is shown at higher magnification in ***A′***, including orthogonal sections to demonstrate TH immunolabeling within RABV-GFP-positive NG cells. Scale bar: ***A′***, 10 μm. ***B***, RNAscope for *Gad1* mRNA (red) and immunolabeling for GFP (green) and TH (cyan) in the AP 7 d after inoculation with (EnvA)-RABV-ΔG-GFP into the cNTS. Scale bar, 200 μm. ***B***, Boxed area is shown at higher magnification in ***B′***. Arrows filled with white indicate neurons double-positive for *Gad1* mRNA and RABV-GFP. Arrows filled with red indicate neurons double-positive for TH and RABV-GFP. Scale bar, 50 μm.

Having identified central sources of direct input to the mouse DVC, as well as sources of monosynaptic and/or polysynaptic input specifically to PPG neurons within the cNTS, we next set out to unequivocally determine which of these inputs are monosynaptic. To this end, we bilaterally injected a mixture of Cre-dependent helper viruses (AAV8/733-FLEX-RabiesG and AAV5-FLEX-TVA:mCherry; Table 1) into the cNTS of *mGlu-Cre/tdRFP* transgenic mice (Fig. 9*A*). The same mice underwent a second stereotaxic surgery 24 d later, when the cNTS was targeted unilaterally with EnvA-pseudotyped G-deleted RABV encoding GFP [(EnvA)-RABV-ΔG-GFP; Table 1]. Seven days after infection with RABV, starter neurons (dsRed/GFP-double-positive) were observed in the cNTS (Fig. 9*B*). The cNTS contained additional cells immunopositive only for GFP, consistent with results from the PRV-Introvert-GFP tracing experiment. While these results support the existence of local NTS neural inputs to PPG neurons, some local GFP expression could arise from low levels of leaky expression of TVA, potentially permitting RABV to enter non–Cre-expressing neurons within the RABV injection site (Miyamichi et al., 2013; Beier et al., 2015); however, since these neurons cannot replicate RABV in the absence of Cre-mediated G expression, they cannot serve as starter neurons. Interestingly, while GFP-positive neurons were observed within the IRT (again, consistent with the PRV-Introvert-GFP results), these did not include PPG neurons (Fig. 9*C*), evidence that PPG neurons in IRT do not provide synaptic input to PPG neurons in cNTS. This result also indicates that GFP labeling observed in other locations (Fig. 10) originated from starter PPG neurons within the cNTS, not the IRT. Sources of direct, monosynaptic input to cNTS PPG neurons included the NG, AP, Bar, gigantocellular nucleus, RMg, ventrolateral periaqueductal gray, PSTh, LH, lateral CeA, PVN, and anterior dorsolateral BST (Fig. 10; Table 5) as well as the spinal cord (data not shown). These areas largely correspond to regions that contained GFP-positive cells 96 h after PRV-Introvert-GFP was injected into the cNTS (Figs. 6, 8; Table 5). Exceptions included GFP immunolabeling observed within the RMg and RPa after RABV injection (and CTb labeling in the same regions), but no GFP labeling derived from PRV-Introvert-GFP at the 96 h postinoculation time point (Table 5); however, PRV-GFP labeling was observed in RMg and RPa at the 120 h postinoculation time point. Another exception was GFP labeling derived from PRV-Introvert-GFP in the Sp5 at the 96 h time point, but no GFP labeling in the Sp5 derived from RABV tracing (Table 5). Many of the NG vagal sensory neurons identified by monosynaptic RABV tracing as providing direct synaptic input to cNTS PPG neurons were TH-positive (Fig. 11*A*). Within the AP, some GFP-positive cells were *Gad1* or TH-positive (Fig. 11*B*).

### Experiment 4: DVC-projecting neurons in Bar, PVN, and LH are activated in mice after acute restraint stress

**Figure 12.**
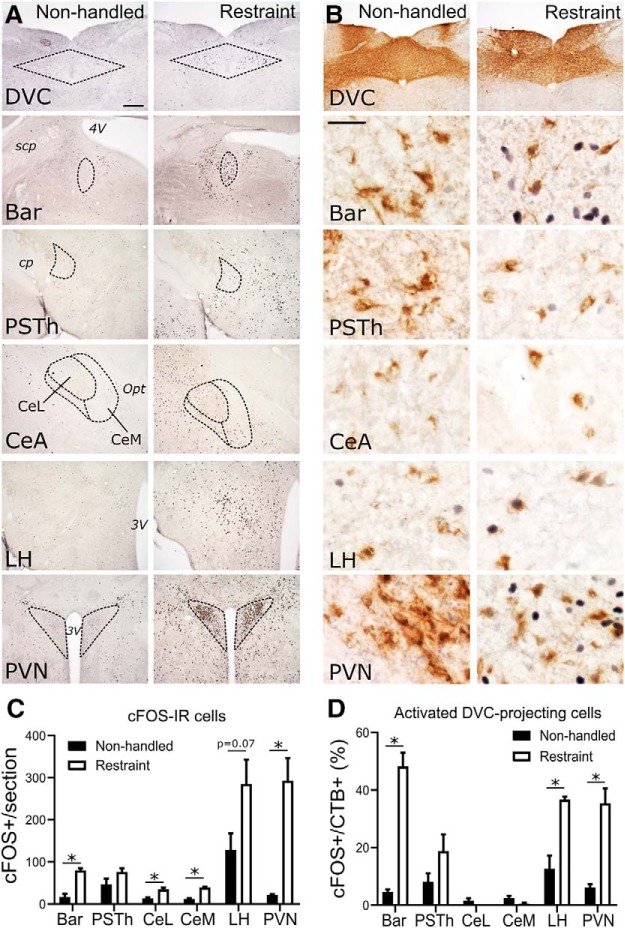
Acute restraint stress activates CTb-labeled, DVC-projecting neurons in Bar, LH, and PVN. ***A***, cFOS-immunoreactive (IR) cells (black cell nuclei) in cNTS (DVC), Bar, PSTh, CeA, LH, and PVN in mice perfused 60 min after the end of 30 min restraint stress. Scale bar: ***A***, 200 μm (applies to all panels). ***B***, cFOS-IR (black nuclei) and CTb-IR (brown cytoplasmic labeling) in DVC (injection site), Bar, PSTh, CeA, LH, and PVN in mice exposed to 30 min restraint stress. Scale bar: All panels except top, 30 μm. ***C***, cFOS-IR cell counts (group mean ± SEM) in nonhandled control mice (*n* = 5) versus mice exposed to restraint stress (*n* = 4) in the following brain regions: Bar (*p = 0.0018), PSTh (*p* = 0.15), CeL (*p = 0.013), CeM (*p = 0.0008), LH (*p* = 0.0677), and PVN (*p = 0.0053). ***D***, Calculated percentage of CTb-labeled cells (group mean ± SEM) that were also cFOS-IR in nonhandled mice (*n* = 5) versus mice exposed to 30 min restraint stress (*n* = 4) in Bar (*p = 0.0001), PSTh (*p* = 0.16), CeL (*p* = 0.25), CeM (*p* = 0.16), LH (*p = 0.006), and PVN (*p = 0.0015). ***C***, ***D***, *p* values from multiple unpaired Student's *t* tests, corrected using false discovery rate. Abbreviations defined in Table 4.

Since PPG neurons are activated to express cFOS in rats and mice after acute psychogenic stress (i.e., restraint), we next asked which of the brain regions identified as sources of synaptic input to cNTS PPG neurons are activated in mice after acute restraint stress. Five brain regions (Bar, PSTh, LH, PVN, and CeA) displayed particularly robust GFP labeling in mice after either PRV-Introvert-GFP (Figs. 6, 8) or monosynaptic RABV tracing (Fig. 10). Both PRV and RABV infection alters host cell gene expression (Weiss and Chowdhury, 1998; Prosniak et al., 2001; Ray and Enquist, 2004; Skiba et al., 2010; Gomme et al., 2012), precluding their combination with cFOS analyses. Thus, mice that received DVC/cNTS iontophoretic delivery of CTb were used in this experiment. Mice that were exposed to restraint stress displayed significantly more cFOS-positive neurons within Bar, PVN, and CeA (both lateral and medial subnuclei) compared with nonhandled controls (Fig. 12*A*,*C*), and tended to have higher numbers of cFOS-positive neurons in the LH, although this difference (compared with nonstressed control mice) did not reach statistical significance (*p* = 0.07). Significantly larger proportions of CTb-positive (i.e., DVC-projecting) neurons in Bar, LH, and PVN were activated after acute stress compared with the control condition (Fig. 12*B*,*D*). Thus, these regions are potential sources of input promoting stress-induced activation of DVC neurons, which are known to include PPG neurons in the mouse cNTS (Terrill et al., 2019). However, although restraint stress increased cFOS labeling within the medial and lateral CeA (Fig. 12*A*,*C*), the cFOS-positive CeA neurons were not CTb-positive (Fig. 12*B*,*D*). Thus, CeA neurons that project to the DVC, which are GABA-ergic in rats (Saha et al., 2000), are not activated to express cFOS in mice after restraint stress.

### Bar Crh neurons provide monosynaptic input to cNTS PPG neurons

**Figure 13.**
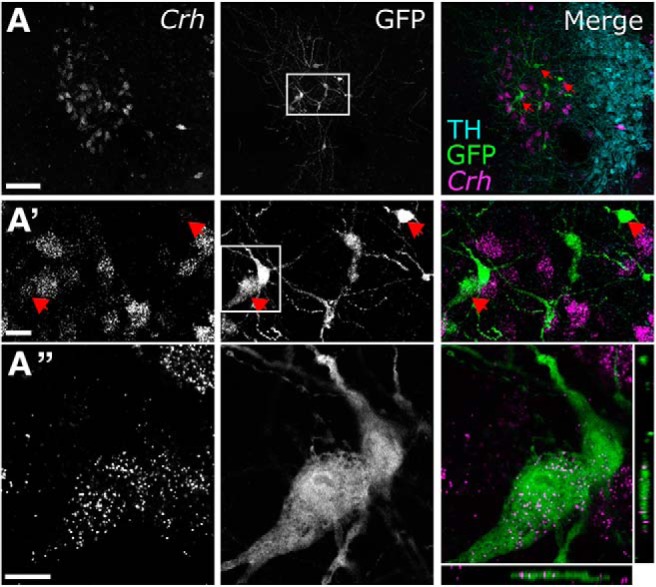
Chemical phenotype of RABV-infected Bar neurons providing monosynaptic input to cNTS PPG neurons. ***A***, RNAscope for *Crh* mRNA (magenta) and immunoreactivity for GFP (green) and TH (cyan) in Bar and LC 7 d after inoculation with (EnvA)-RABV-ΔG-GFP into the cNTS. TH immunolabeling identifies the LC, and is shown only in the merged image for anatomical orientation. Scale bar: ***A***, 100 μm. ***A′***, Higher-magnification views of the boxed region indicated in ***A*** (top row). Red arrows indicate neurons double-positive for *Crh* mRNA and GFP. Scale bar, 20 μm. ***A″***, Higher-magnification view of the boxed region in ***A′*** (GFP), including orthogonal sectioning to demonstrate *Crh* mRNA within GFP-positive cells in Bar. Scale bar, 10 μm.

Given that DVC-projecting neurons within Bar were particularly responsive to restraint stress (Fig. 12*B*,*D*), we used RNAscope ISH to identify the molecular phenotype of Bar neurons that provide synaptic input to cNTS PPG neurons. Bar neurons in mice are glutamatergic, and a subset express Crh (Pavcovich and Valentino, 1995; Hou et al., 2016; Peng et al., 2017; Verstegen et al., 2019). Consistent with this, we found that *Crh* mRNA was expressed by a subset of Bar neurons providing direct input to PPG neurons, as evidenced by GFP reporter expression after monosynaptic RABV tracing (Fig. 13).

## Discussion

**Figure 14.**
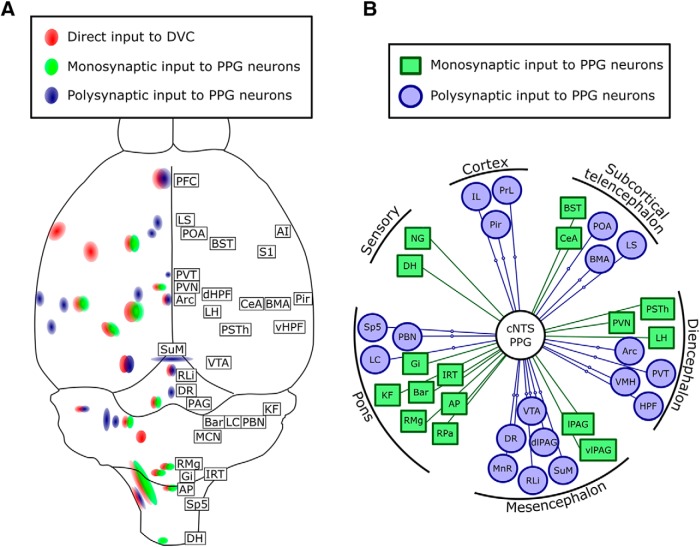
Two summary schematics (***A***, ***B***) indicating central sources of axonal input to the DVC (red, retrograde CTb labeling), sources of monosynaptic input to cNTS PPG neurons (green, RABV labeling and PRV labeling), and sources of indirect (i.e., polysynaptic) input to NTS PPG neurons (blue, PRV labeling but no RABV labeling).

Visceral sensory-motor signal processing within the DVC is controlled by spinal, brainstem, hypothalamic, and limbic forebrain regions that modulate responses to homeostatic and psychogenic challenges (Rinaman, 2011; Grill and Hayes, 2012; Maniscalco and Rinaman, 2018). The present report is the first to describe central sources of neural input to the DVC in mice, and the first to use a side-by-side comparison of PRV and RABV tracing to identify sources of monosynaptic and polysynaptic input to genetically defined neurons. We find that direct inputs to the mouse DVC are similar to those reported for rats (van der Kooy et al., 1984; Veening et al., 1984; Berthoud et al., 1990; Ruggiero et al., 1996), and that cNTS PPG neurons in mice receive monosynaptic input from vagal sensory neurons in the NG, from the spinal cord dorsal horn, and from forebrain, midbrain, and hindbrain regions implicated in the central control of autonomic function, motivated behavior, and stress responses. The anatomical organization of monosynaptic and polysynaptic inputs to the mouse DVC, and specifically to cNTS PPG neurons, is summarized in Figure 14. We also identify the molecular phenotype of some of the monosynaptic inputs to PPG neurons (i.e., TH-positive neurons within the NG and AP, and *Crh*-expressing neurons in Bar), and reveal novel polysynaptic connections from both the hippocampal formation and the paraventricular thalamus to cNTS PPG neurons. Finally, we demonstrate that DVC-projecting neurons within the LH, PVN, and Bar are activated to express cFOS in mice exposed to acute restraint stress, highlighting these projections within a larger network of distributed neural circuits that orchestrate physiologic and behavioral stress responses.

### Central input to the DVC in the mouse

In addition to their vital role in processing visceral sensory signals and modulating vagal motor outflow (Rogers et al., 1995; Grill and Hayes, 2012), NTS neurons modulate hypothalamic neuroendocrine secretion, food intake, and other motivated behaviors (Maniscalco and Rinaman, 2018; Rinaman, 2010, 2019). Indeed, food intake is suppressed in mice after chemogenetic activation of specific subpopulations of NTS neurons, including those expressing CCK (D'Agostino et al., 2016; Roman et al., 2017), 5-HT_2C_ receptor (D'Agostino et al., 2018), PPG (Gaykema et al., 2017; Liu et al., 2017; Holt et al., 2019), POMC (Zhan et al., 2013), dopamine β hydroxylase (Roman et al., 2016), and Phox2b (Wang et al., 2015). Subpopulations of NTS neurons respond to interoceptive vagal sensory signals (Vrang et al., 2003; Appleyard et al., 2007; Hayes et al., 2009; Grill and Hayes, 2012) and to cognitive signals (Ulrich-Lai and Herman, 2009; Furlong et al., 2014; Maniscalco et al., 2015; Myers et al., 2017). Our CTb tracing data in mice indicate that the DVC (which includes the NTS, AP, and DMV) receives direct input from cortical and subcortical telencephalic regions, as well as the hypothalamus, midbrain, pons, hindbrain, and spinal cord (Table 5; Fig. 14*A*), consistent with evidence in rats (van der Kooy et al., 1984; Veening et al., 1984; Berthoud et al., 1990; Ruggiero et al., 1996).

### Technical considerations

Although CTb injection sites were targeted to the cNTS, they extended into the adjacent DMV and AP; thus, we conservatively describe retrograde CTb labeling from these injection sites as evidence for neural inputs to the DVC. Moreover, CTb is taken up not only by nerve terminals, but also by fibers of passage (Luppi et al., 1990). Despite this potential complication, the fact that the vast majority of the central regions that contained CTb retrograde labeling were also labeled in the PRV and RABV tracing experiments (discussed further, below) provides evidence for direct synaptic contacts within the cNTS. In this regard, PRV is taken up preferentially by synaptic terminals and is strictly retrogradely transported (Card et al., 1993; Pickard et al., 2002; Card and Enquist, 2014; Pomeranz et al., 2017). Thus, when central inputs specific to PPG neurons were revealed using PRV-Introvert-GFP, infected “starter neurons” presumably included only PPG neurons with axon terminals present within the DVC injection site. Conversely, RABV is readily taken up by neuronal cell bodies as well as by axon terminals within the CNS (Ugolini, 2008). The comparable labeling patterns observed after PRV-Introvert or RABV injection indicate that cNTS PPG neurons give rise to local axon collaterals within the DVC, consistent with our recent report in both rats and mice (Card et al., 2018).

There were a few differences in results obtained from RABV tracing and from PRV infection at the 96 h postinoculation interval (Table 5). Two raphe nuclei, the RMg and RPa, contained scattered GFP-positive cells following RABV tracing and also contained CTb retrograde labeling, whereas PRV-induced GFP labeling in RMg and RPa was absent at 96 h (but present by 120 h). While the appearance of PRV-GFP labeling at the longer time point by itself might be interpreted as evidence against direct monosynaptic inputs to PPG neurons, the RABV-GFP labeling indicates that monosynaptic inputs do exist. It is possible that the “delay” in PRV-GFP labeling is due to a low density of synaptic inputs to PPG neurons, such that additional time is necessary to establish productive PRV-GFP infection (Card et al., 1999; Card and Enquist, 2014). Another difference was that the Sp5 was labeled in both CTb tracing and in PRV short postinoculation interval (96 h) experiments, but not after RABV tracing. We interpret these results as evidence that neurons in the Sp5 innervate target neurons within the DVC that provide synaptic input to PPG neurons. Importantly, these interesting and potentially meaningful differences in tracing data were only revealed as a result of both PRV and RABV tracing, highlighting the interpretive value of comparing results obtained from using these two complimentary techniques in parallel experiments.

### cNTS PPG neurons receive local synaptic input from other DVC neurons

Non-PPG neurons within the cNTS and AP were infected with PRV at early postinoculation intervals. PRV-infected GFP-positive cNTS neurons were TH-negative, whereas RABV-infected, GFP-labeled AP neurons were TH-positive. Thus, local synaptic inputs to cNTS PPG neurons include noradrenergic and/or dopaminergic AP neurons, but not cNTS noradrenergic neurons of the A2 cell group.

### Monosynaptic inputs to PPG neurons arise from the NG and central nuclei implicated in autonomic control and stress responses

In addition to local inputs from neurons within the DVC, monosynaptic inputs to cNTS PPG neurons originated from the NG, spinal cord, and many brain regions. Inputs from vagal sensory NG neurons are consistent with a report that PPG neurons in mouse *ex vivo* slices are excited in response to electrical stimulation of the solitary tract (Hisadome et al., 2010). Direct vagal afferent input to PPG neurons provides an anatomical basis for GLP-1 neural responsiveness to interoceptive vagal stimuli in rats, including gastric distension (Vrang et al., 2003; Hayes et al., 2009) and systemic lithium chloride (Rinaman, 1999) or CCK (Rinaman, 1999; Maniscalco and Rinaman, 2013). Lithium chloride also activates GLP-1 neurons in CD-1 mice (Lachey et al., 2005), but effects of gastric distension or systemic CCK on PPG/GLP-1 neurons in mice have not been reported.

In addition to direct vagal sensory input, we provide the first evidence that cNTS PPG neurons receive direct input from the spinal cord, consistent with the spinosolitary tract described in rats (Menétrey and Basbaum, 1987; Menétrey and De Pommery, 1991). The spinosolitary tract integrates somatic and visceral sensory inputs (Cervero, 1994), and has been implicated in the integration of visceral and somatosensory nociception. Thus, spinal inputs to cNTS PPG neurons could convey nociceptive signals and/or could coordinate somatic and autonomic motor functions.

RABV tracing indicated that cells within the medullary reticular formation, including the IRT and RMg, are monosynaptically linked to cNTS PPG neurons. Interestingly, labeled neurons within the IRT did not include PPG neurons, suggesting that this adjacent population of PPG neurons does not provide synaptic input to cNTS PPG neurons. Labeled neurons within RMg are likely serotonergic (not confirmed here), which would be consistent with reports that serotonin modulates mouse PPG neuron activity *in vitro*, and that serotonin-induced hypophagia depends on central GLP-1 signaling (Holt et al., 2017; Leon et al., 2019).

Regarding higher regions of the brainstem and forebrain, the PVN, LH, CeA, PSTh, and Bar are the most prominent sources of monosynaptic input to cNTS PPG neurons, evidenced by robust GFP labeling in mice after RABV injection and in mice killed 72 h after injection of PRV-Introvert-GFP. All of these regions have been implicated in the central control of food intake, autonomic outflow, and behavioral and physiologic responses to stress (Sved et al., 2002; Ciriello et al., 2008; Ulrich-Lai and Herman, 2009; Seoane-Collazo et al., 2015; Arrigoni et al., 2019; Ip et al., 2019). Interestingly, while the majority of CeA input to the DVC arises from the medial CeA subnucleus, CeA neurons supplying direct synaptic input to cNTS PPG neurons preferentially occupy the lateral CeA. Several genetically defined subpopulations of neurons within the lateral CeA have been shown to modulate feeding in mice (Cai et al., 2014; Douglass et al., 2017; Hardaway et al., 2019), and the CeA is critical for the suppression in food intake in rats in response to threats (Petrovich et al., 2009). It therefore seems plausible that the lateral CeA participates in stress-induced hypophagia by modulating the activity of cNTS PPG neurons.

By comparing CTb retrograde tracer labeling with GFP immunolabeling patterns after monosynaptic RABV tracing and after PRV tracing at different postinoculation intervals, it is possible to create a map of monosynaptic and disynaptic inputs to cNTS PPG neurons (Fig. 14*A*,*B*). Our results indicate that areas, including the hippocampal formation, basomedial amygdala, ventral tegmental area, and paraventricular thalamus, contain neurons that polysynaptically impinge on cNTS PPG neurons. These results highlight potential pathways through which PPG neural activity can be modulated, with resulting effects on food and water intake, drug reward, as well as autonomic outflow and other stress-related functions (Ghosal et al., 2013; Skibicka, 2013; Hayes and Schmidt, 2016; Holt and Trapp, 2016).

### Stress-induced activation of PVN, LH, and Bar neural inputs to the DVC/cNTS

Many of the brain regions providing synaptic input to PPG neurons in the cNTS have been implicated in stress responsiveness. Thus, we examined whether acute stress activates central neural inputs to the DVC/cNTS in mice, which could underlie stress-induced activation of PPG/GLP-1 neurons (Maniscalco et al., 2015; Terrill et al., 2019). Restraint stress-induced cFOS was quantified in a subset of brain areas that displayed monosynaptic RABV-induced GFP labeling in other mice. The results indicate that DVC-projecting cells within the PVN, LH, and Bar are activated in mice exposed to acute restraint stress. In rats, DVC-projecting cells within the PVN also are activated to express cFOS after acute stress (Dayas et al., 2004; Furlong et al., 2014), whereas stress-induced activation of DVC-projecting LH and Bar neurons has not previously been reported. Interestingly, while unspecified CeA neurons are activated to express cFOS following stress, DVC-projecting CeA neurons are not, suggesting that stress removes this source of inhibitory input to PPG neurons, since CeA neurons that target the mouse DVC presumably are GABAergic, as in rats (Saha et al., 2000).

Our finding that DVC-projecting Bar neurons are particularly stress-sensitive is intriguing, considering the role of Bar in modulating motor functions of the colon, bladder, and other pelvic viscera (Pavcovich and Valentino, 1995; Cano et al., 2000; Sved et al., 2002; Hou et al., 2016; Verstegen et al., 2019). Bar is located just medial to the LC (Pavcovich and Valentino, 1995; Cano et al., 2000; Hou et al., 2016), and is often mistaken for the LC or the adjacent dorsal tegmental nucleus (Sved et al., 2002). RNAscope ISH revealed that some Bar neurons projecting directly to cNTS PPG neurons express *Crh*, evidence that these neurons supply a portion of the known CRH input to the cNTS (Veening et al., 1984; Sawchenko, 1987; Wang et al., 2018, 2019). In addition to Bar, other known sources of CRH input to the DVC (Peng et al., 2017) contained neurons that were monosynaptically labeled after RABV tracing from PPG neurons; these included the PVN, CeA, BST, and PAG. Future studies will determine the molecular phenotypes of these and other monosynaptic inputs.

In conclusion, this report provides evidence that PPG/GLP-1 neurons within the cNTS receive direct synaptic input from vagal sensory neurons, spinal dorsal horn neurons, and neurons within multiple brainstem and forebrain regions. These inputs define a widely distributed neural network through which interoceptive, exteroceptive, and cognitive stimuli can modulate the activity of PPG neurons, providing routes for stress-induced recruitment of central GLP-1 signaling pathways that impact motivated behavior, emotional state, neuroendocrine secretion, and autonomic outflow (Kinzig et al., 2003; Maniscalco et al., 2015; Ghosal et al., 2017; Williams et al., 2018; Terrill et al., 2018, 2019; Holt et al., 2019; Zheng et al., 2019). Within this context, our new findings point to the PVN, LH, and Bar as three sources of input that may drive stress-induced activation of PPG neurons. Future studies should investigate the necessity and sufficiency of these circuits in mediating behavioral and physiologic responses to stress.
